# Comparison of Rates of Lower Extremity Amputation in Patients With and Without Gout in the US Department of Veterans Affairs Health System

**DOI:** 10.1001/jamanetworkopen.2021.42347

**Published:** 2022-01-06

**Authors:** Ted R. Mikuls, Quint Soto, Alison Petro, Lindsay Helget, Punyasha Roul, Harlan Sayles, Brendan Cope, Tuhina Neogi, Brian LaMoreaux, James R. O’Dell, Bryant R. England

**Affiliations:** 1 Veterans Affairs Nebraska–Western Iowa Health Care System, Omaha, Nebraska; 2 Division of Rheumatology, Department of Internal Medicine, University of Nebraska Medical Center, Omaha; 3 Department of Biostatistics, College of Public Health, University of Nebraska Medical Center, Omaha; 4 Section of Rheumatology, Department of Medicine, Boston University School of Medicine, Boston, Massachusetts; 5 Horizon Therapeutics, Deerfield, Illinois

## Abstract

**Importance:**

Cardiometabolic and other risk factors could render patients with gout more likely to undergo lower extremity amputation (LEA).

**Objective:**

To examine the rate of and factors associated with LEA in patients with gout.

**Design, Setting, and Participants:**

In this matched cohort study using national administrative data, multivariable Cox proportional hazards regression models were used to examine the associations of gout with LEA. In analyses limited to patients with gout, attributes of serum urate control and treatment with urate-lowering therapy were examined as factors associated with LEA. This study included patients who used US Department of Veterans Affairs services from January 1, 2000, to July 31, 2015. Patients with gout were identified using diagnostic codes and matched with up to 10 controls by age, sex, and year of benefit enrollment. Data analysis was performed from January 26, 2021, to September 3, 2021.

**Exposures:**

Gout classification served as the primary independent variable of interest. In analyses limited to patients with gout, factors associated with serum urate control and urate-lowering therapy were examined.

**Main Outcomes and Measures:**

Overall LEA, as well as toe, transmetatarsal, below-the-knee, and above-the-knee amputation.

**Results:**

This cohort study included 5 924 918 patients, 556 521 with gout (mean [SD] age, 67 [12] years; 550 963 (99.0%) male; 88 853 [16.0%] Black non-Hispanic; 16 981 [4.3%] Hispanic/Latinx; 345 818 [62.1%] White non-Hispanic; 80 929 [14.5%] with race and ethnicity data missing; and 23 940 [4.3%] classified as other) and 5 368 397 without gout (mean [SD] age, 67 [12] years; 5 314 344 [99.0%] male; 558 464 [10.4%] Black non-Hispanic; 204 291 [3.0%] Hispanic/Latinx; 3 188 504 [59.4%] White non-Hispanic; 1 257 739 [23.4%)] with race and ethnicity data missing; and 159 399 [3.0%] classified as other). Compared with patients without gout, patients with gout were more likely to undergo amputation, an increased rate that remained after adjustment (adjusted hazard ratio, 1.20; 95% CI, 1.16-1.24) and was highest for below-the-knee amputation (adjusted hazard ratio, 1.59; 95% CI, 1.39-1.81). In those with gout, poor serum urate control (mean >7 mg/dL during the preceding year) was associated with a 25% to 37% increase in the rate of amputation. In contrast, treatment with urate-lowering therapy was not associated with the LEA rate.

**Conclusions and Relevance:**

In this matched cohort study, patients with gout were more likely to undergo LEA. This increase was independent of other comorbidities that have been associated with amputation, including diabetes and peripheral vascular disease. Serum urate control was independently associated with the LEA rate, suggesting the possibility that lower extremity amputation may be preventable in some patients.

## Key Points

**Question:**

Are lower extremity amputations (LEAs) performed more often in patients with gout?

**Findings:**

In this cohort study of 5 924 918 patients with and without gout, patients with gout had higher rates of LEA than those without gout after adjusting for comorbidities and other risk factors; this increase was apparent across different types of LEA. Among those with gout, poor serum urate control, but not the administration of urate-lowering therapy, was associated with a higher incidence of LEA.

**Meaning:**

These findings suggest that because gout is associated with an increased rate of LEA, efforts to better understand the degree to which these procedures might be preventable is warranted.

## Teaser

This cohort study of patients from the US Department of Veterans Affairs Health System compares the rates of lower extremity amputations in patients with and without gout, as well as factors that might be associated with differences.

## Introduction

Gout is the most common form of inflammatory arthritis worldwide, affecting up to 4% of all adults with higher prevalence rates in older men and in members of minoritized racial and ethnic minority groups.^1^ Characterized by hyperuricemia and articular deposition of monosodium urate, the clinical course of gout consists of painful arthritis flares separated by asymptomatic intercritical periods of variable duration. In the absence of effective urate-lowering therapy (ULT), flare frequency and severity increase over time, with some patients developing advanced gout characterized by tophi and chronic systemic inflammation. As such, gout poses a substantial and increasing burden, resulting annually in more than 200 000 emergency department visits^2,3^ and total health care costs exceeding $6 billion in the US alone.^4^ A significant proportion of costs are preventable, reflecting missed treatment opportunities. With an increasingly well-understood pathogenesis, gout represents a highly treatable condition with management strategies focused on anti-inflammatory agents for flares and ULT to reduce or even halt disease progression.^5,6^

Although gout alone poses a substantial burden, it also has strong associations with comorbidities and increased mortality. Cardiovascular disease, hypertension, chronic kidney disease (CKD), and diabetes are all overrepresented in patients with gout.^7^ Compared with the general population, patients with gout demonstrate a greater than 25% increase in the rate of acute myocardial infarction^8^ and are more likely to develop diabetes.^9^ Notably, these comorbidities also portend an increased risk of undergoing lower extremity amputation (LEA). Diabetes, for example, is associated with a 6-fold greater likelihood of receiving LEA, whereas advanced forms of CKD portend between a 2- to 4-fold increase in the rate of amputation.^10^

In addition to concomitant comorbidity and potential adverse effects of serum urate and inflammation on vascular function,^11,12,13^ arthritis manifestations might also influence LEA rates in gout. It is recognized, for example, that gout mimics infectious processes of septic arthritis,^14^ cellulitis,^15^ and osteomyelitis^16^ and can masquerade as a nonhealing diabetic foot ulcer,^17^ which is a frequent indication for LEA.^18^ Numerous reports^18,19,20,21,22^ have described digital and/or lower limb amputations in the context of gout, some^21,22^ citing infection in the setting of gout, and another^19^ reported a gout diagnosis made postoperatively after histopathologic examination of resected tissues. Whether patients with gout undergo LEA at a higher rate than those without gout is unknown.^23^ Addressing this knowledge gap and identifying LEA determinants would inform future efforts to reduce amputations and improve outcomes in gout.

The goal of this study was to examine the rate of and factors associated with LEA in patients with gout. To begin to understand the degree to which LEA might be preventable, we also examined whether amputation in gout was associated with measures of serum urate control or ULT administration. We hypothesized that patients with gout would have higher rates of LEA than patients without gout, independent of comorbidity and other risk factors, and among those with gout, higher serum urate concentrations and suboptimal ULT would be associated with increased amputation rates.

## Methods

This national, matched cohort study was approved by the institutional review board at the Veterans Affairs (VA) Nebraska–Western Iowa Health Care System. This study involved the use of identifiable data. The requirement for informed consent was waived per institutional policy because there was no prospective data collection or patient contact. This report adheres to the Strengthening the Reporting of Observational Studies in Epidemiology (STROBE) reporting guideline for cohort studies.^24^

### Study Population and Data Source

This study leveraged administrative data from the national Veterans Health Administration (VHA). We used inpatient and outpatient data in the VA Corporate Data Warehouse, accessed through the VA Informatics and Computing Infrastructure. Data elements included pharmacy-dispensing, laboratory data, *Current Procedural Terminology, Fourth Edition (CPT-4)* and *International Classification of Diseases, Ninth Revision (ICD-9)* codes corresponding to electronic health record data from January 1, 2000, through July 31, 2015. We limited cohort creation to avoid misclassification related to *International Statistical Classification of Diseases and Related Health Problems, Tenth Revision (ICD-10)* implementation initiated in October 2015. We identified patients with gout using an algorithm that required patients to have an *ICD-9* code of 274.xx from 2 or more encounters 30 or more days apart with the second date serving as the index date.^25^ Each case was then matched with up to 10 patients without gout by birth year, sex, and year of VHA enrollment (the latter to account for temporal health care trends^10^ and to ensure similar durations of preindex observation). Comparators without gout had no prior *ICD-9* codes of gout or receipt of previous ULT (allopurinol, febuxostat, probenecid, or pegloticase) and were assigned the same index date as their matched case counterpart. Potential cases and controls were excluded if they received LEA in the VHA before the index date. Study participants were followed up from the index date until first LEA, death, or the end of follow-up (September 2015), whichever occurred first. Some patients without gout were censored at the time they fulfilled the gout algorithm, after which they were crossed over and contributed to gout observation after matching with up to 10 new controls. Data analysis was performed from January 26, 2021, to September 3, 2021.

### Lower Extremity Amputation 

Lower extremity amputation was defined using a combination of *CPT4, ICD-10* Procedure Coding System, and *International Classification of Diseases, Ninth Revision, Clinical Modification (ICD-9-CM)* codes as described by Cai et al^10^ (eTable 1 in the Supplement). Only the first LEA event was examined to reduce bias associated with multistage amputation procedures or revisions. Procedures were further classified by type: toe, transmetatarsal, below the knee, or above the knee. Crude incidence rates (IRs) of LEA and 95% CIs were calculated in patients with gout and comparators without gout by dividing the number of events by the total patient-years of follow-up in each group.

### Suboptimal Serum Urate Control and ULT

To examine associations of gout care with LEA, we examined 2 components of surveillance and management during each year of follow-up among patients with gout: the receipt of conventional oral ULT and serum urate concentration. Using an a priori definition, patients were conservatively considered to have received adequate ULT with 2 or more pharmacy dispensing-episodes together totaling at least 90 days of coverage with allopurinol, febuxostat, or probenecid within each 1-year follow-up interval. Individuals receiving none of these agents were considered to have received suboptimal ULT. Those dispensed ULT during the year but not satisfying criteria for adequate treatment were categorized as indeterminate. Using an a priori definition for serum urate measures, patients with mean urate values less than 6 mg/dL (to convert to millimoles per liter, multiply by 0.0595) during each year of follow-up were considered to have adequate serum urate control, consistent with gout management guidelines,^5,6^ whereas those with mean levels greater than 7 mg/dL were considered to have suboptimal control. Missing values for a given year were imputed using available values for up to 2 prior years, recognizing that patients with well-controlled gout may undergo infrequent testing and that testing frequency in a real-world setting is variable. Patients with gout with serum urate levels of 6 to 7 mg/dL or for whom no serum urate measure was available were categorized as having indeterminate serum urate control.

### Statistical Analysis

Group characteristics were compared using a 2-tailed, unpaired *t* test for continuous variables or a χ^2^ test for categorical variables. In primary analyses, the association of gout with LEA was examined using unadjusted Cox proportional hazards regression followed by multivariable Cox proportional hazards regression. Covariates included in multivariable models were selected a priori, were ascertained using administrative and electronic health data, and included age, sex, race, ethnicity, body mass index (calculated as weight in kilograms divided by height in meters squared), smoking history, hypertension, cardiovascular disease, peripheral artery disease, cancer, cerebrovascular disease, chronic lung disease, dementia, diabetes, and kidney disease. Race and ethnicity information was taken from electronic health care data and included as a covariate given the variability in gout prevalence and comorbidities across categories. Race and ethnicity reported as other included American Indian or Alaska Native, Asian, multiple race, or Native Hawaiian or other Pacific Islander. Body mass index was calculated from the closest weight value preceding the index date and modal height within the electronic health record. Smoking information was collected from routine clinical notes (VHA health factors) and modeled as current, former, never, or missing, with missing included because missing values may be informative (eg, death prohibiting medical record documentation). Comorbidities were defined based on the presence of at least 2 corresponding *ICD-9* or *ICD-9-CM* codes occurring at any time before the index date (eTable 2 in the Supplement). The association of gout with LEA was quantified using hazard ratios (HRs) and 95% CIs, where the cause-specific hazard is interpreted as the hazard of amputation while the patient is alive and free of LEA. Two additional sensitivity analyses were conducted to evaluate missing covariates or loss of follow-up. Specifically, associations of gout with overall LEA were examined after removing individuals with missing data for race and ethnicity or smoking (ie, complete case approach) and censoring patients with more than 365 consecutive days without active VHA follow-up (defined by the occurrence of an outpatient visit or hospitalization).

Recognizing diabetes as the strongest factor associated with LEA occurrence among US veterans,^10^ we subsequently examined patients in 4 groups defined by the presence or absence of gout and diabetes as part of preplanned secondary analyses, again using Cox proportional hazards regression models and the aforementioned covariates. To explore the degree to which amputation events might be associated with factors related to gout-specific health care, we examined components of surveillance and management as determinants of LEA in analyses restricted to patients with gout. Those with adequate or indeterminate care were combined into a single group for ease of interpretation. Patients were then categorized during each year of follow-up into 1 of 4 groups based on these 2 components of gout care: (1) adequate or indeterminate serum urate control and adequate or indeterminate ULT (Reference group), (2) adequate or indeterminate serum urate control and suboptimal ULT, (3) suboptimal serum urate control and adequate or indeterminate ULT, or (4) suboptimal serum urate control and suboptimal ULT. Associations of this combined variable with LEA during the next year of follow-up were examined using multivariable Cox proportional hazards regression models as described above, with the index calendar year serving as an additional covariate in each model (eFigure in the Supplement). Note that the serum urate and ULT status variable could change from year to year within a patient, but all other covariates were fixed index values. All analyses were performed using Stata software, version 15 (StataCorp) within the VA Informatics and Computing Infrastructure. A 2-tailed *P* < .05 was considered statistically significant.

## Results

This cohort study included 5 924 918 patients, 556 521 with gout (mean [SD] age, 67 [12] years; 550 963 (99.0%) male; 88 853 [16.0%] Black non-Hispanic; 16 981 [4.3%] Hispanic/Latinx; 345 818 [62.1%] White non-Hispanic; 80 929 [14.5%] with race and ethnicity data missing; and 23 940 [4.3%] classified as other) and 5 368 397 without gout (mean [SD] age, 67 [12] years; 5 314 344 [99.0%] male; 558 464 [10.4%] Black non-Hispanic; 204 291 [3.0%] Hispanic/Latinx; 3 188 504 [59.4%] White non-Hispanic; 1 257 739 [23.4%)] with race and ethnicity data missing; and 159 399 [3.0%] classified as other) (Table 1). Compared with patients without gout, individuals with gout were more likely to be Black or African American, to have obesity, and to have comorbidities, with the exception of dementia, which was slightly more frequent in those without gout. In addition to being less likely to have missing data for race and ethnicity, patients with gout were also less likely than those without gout to have missing data for smoking status (31 965 [5.7%] vs 868 838 [16.2%]).

**Table 1.** Characteristics of US Veteran Patients With and Without Gout at Index Date^a^

Characteristic	Gout (n = 556 521)	Without gout (n = 5 368 397)
Demographic characteristics		
Age, mean (SD), y	67 (12)	67 (12)
Sex		
Male	550 963 (99.0)	5 314 344 (99.0)
Female	5558 (1.0)	54 053 (1.0)
Race and ethnicity		
Black non-Hispanic	88 853 (16.0)	558 464 (10.4)
Hispanic/Latinx	16 981 (4.3)	204 291 (3.0)
White non-Hispanic	345 818 (62.1)	3 188 504 (59.4)
Missing	80 929 (14.5)	1 257 739 (23.4)
Other^b^	23 940 (4.3)	159 399 (3.0)
Health factors and comorbidity		
BMI^c^		
<20	4396 (0.8)	76 986 (1.6)
20 to <25	34 073 (6.2)	629 553 (13.2)
25 to <30	154 863 (28.2)	1 753 507 (36.9)
≥30	356 850 (64.9)	2 296 316 (48.3)
Smoking status		
Never	104 112 (18.7)	896 252 (16.7)
Former	219 980 (39.5)	1 739 862 (32.4)
Current	200 464 (36.0)	1 863 445 (34.7)
Missing	31 965 (5.7)	868 838 (16.2)
Hypertension	404 983 (72.8)	2 296 556 (42.8)
Cardiovascular disease	201 227 (36.2)	1 153 239 (21.5)
Peripheral arterial disease	37 372 (6.7)	229 305 (4.3)
Cancer	65 538 (11.8)	480 159 (8.9)
Cerebrovascular disease	36 897 (6.6)	247 523 (4.6)
Chronic lung disease	67 834 (12.2)	496 195 (9.2)
Dementia	3492 (0.6)	40 969 (0.8)
Diabetes	163 386 (29.4)	996 228 (18.6)
Kidney disease	60 365 (10.9)	161 547 (3.0)

Abbreviation: BMI, body mass index (calculated as weight in kilograms divided by height in meters squared).

^a^ Data are presented as number (percentage) of patients unless otherwise indicated. Patients with and without gout were matched on age, sex, and year of Veterans Health Administration enrollment; values of all other variables differed significantly (*P* < .001) by group.

^b^ Other category for race and ethnicity comprised American Indian or Alaska Native, Asian, multiple races, and Native Hawaiian or other Pacific Islander.

^c^ Data for BMI were missing for 618 374 patients (10.4%).

From January 1, 2000, to July 31, 2015, a total of 4970 LEAs were performed during 3.4 million patient-years of follow-up in patients with gout (IR, 1.46 [95% CI, 1.42-1.50] procedures per 1000 patient-years), and 24 583 LEAs were performed during 32.1 million patient-years of follow-up in comparators (IR, 0.77 [95% CI, 0.76-0.78] procedures per 1000 patient-years) (Table 2). Transmetatarsal procedures were the most common procedure, followed in descending order by above-the-knee, toe, and below-the-knee amputations. Crude IRs were universally higher for patients with gout than patients without gout overall and across LEA categories. Results from unadjusted Cox proportional hazards regression are given in Table 2, showing an increased association of LEA with gout both overall (HR, 1.85; 95% CI, 1.80-1.91) and for each procedure type.

**Table 2.** Crude Incidence Rates of Lower Extremity Amputation in Patients With and Without Gout^a^

Event category	No. of events	Incidence per 1000 patient-years (95% CI)	Unadjusted HR (95% CI)
All			
Gout	4970	1.46 (1.42-1.50)	1.85 (1.80-1.91)
Nongout	24 583	0.77 (0.76-0.78)	1 [Reference]
Toe			
Gout	1002	0.29 (0.28-0.31)	2.00 (1.86-2.14)
Nongout	4374	0.14 (0.13-0.14)	1 [Reference]
Transmetatarsal			
Gout	2375	0.70 (0.67-0.73)	1.81 (1.73-1.90)
Nongout	11 736	0.37 (0.36-0.37)	1 [Reference]
Below the knee			
Gout	296	0.09 (0.08-0.10)	2.05 (1.81-2.33)
Nongout	1486	0.05 (0.04-0.05)	1 [Reference]
Above the knee			
Gout	1297	0.38 (0.36-0.40)	1.79 (1.68-1.90)
Nongout	6987	0.22 (0.21-0.22)	1 [Reference]

Abbreviation: HR, hazard ratio.

^a^ Total follow-up of 3.4 million patient-years in 556 521 unique patients with gout and 32.1 million patient-years in 5 368 397 unique patients without gout; median (IQR) follow-up was 5.4 (2.4-9.4) years in patients with gout vs 5.2 (2.3-9.3) years in patients without gout.

**Figure 1.**
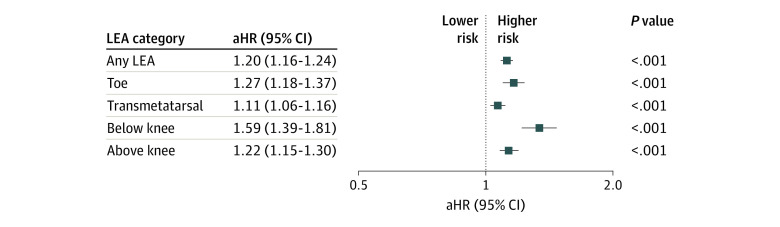
Risk of Undergoing Lower Extremity Amputation (LEA) in Patients With Gout vs Controls Without Gout Adjusted hazard ratios (aHRs) and 95% CIs were generated using multivariable Cox proportional hazards regression models. Estimates provided from separate models examining any LEA (overall) and by LEA type. All models adjusted for age, sex, race, ethnicity, body mass index, smoking history, and comorbidities (hypertension, cardiovascular disease, peripheral artery disease, cancer, cerebrovascular disease, chronic lung disease, dementia, diabetes, and kidney disease).

Results from multivariable Cox proportional hazards regression models are shown in Figure 1. After adjustment, gout remained associated with a significant increase in LEA rate (adjusted HR [aHR], 1.20; 95% CI, 1.16-1.24). Rates were highest for below-the-knee amputations (aHR, 1.59; 95% CI, 1.39-1.81) but were also elevated for toe (aHR, 1.27; 95% CI, 1.18-1.37), transmetatarsal (aHR, 1.11; 95% CI, 1.06-1.16), and above-the-knee (aHR, 1.22; 95% CI, 1.15-1.30) procedures. Results from full models are detailed in eTable 3 in the Supplement. Of other factors examined, the presence of diabetes was most strongly associated with overall LEA (aHR, 3.21; 95% CI, 3.13-3.30). Results for gout were similar in sensitivity analyses using a complete case approach (aHR, 1.18; 95% CI, 1.14-1.22). The association of gout was also similar (aHR, 1.13; 95% CI, 1.09-1.16), albeit slightly attenuated, in sensitivity analyses that censored patients without a VHA observation for 1 year.

**Figure 2.**
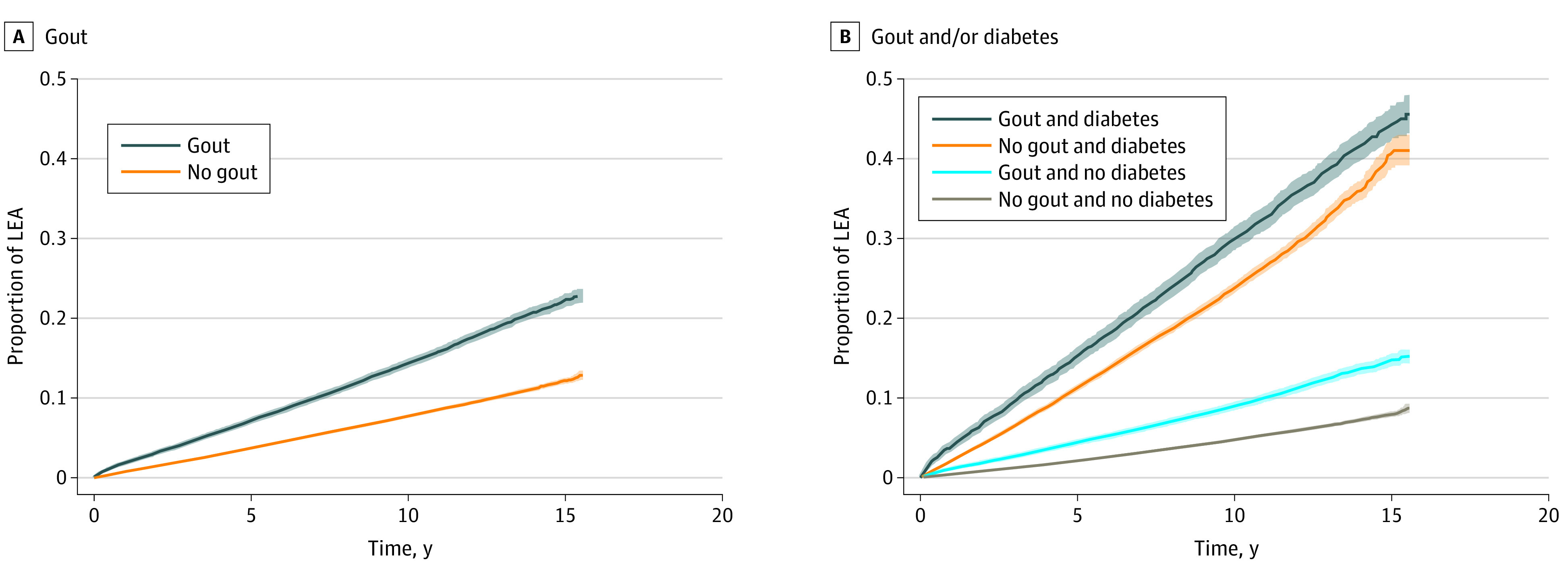
Cumulative Incidence of Undergoing Any Lower Extremity Amputation (LEA) Cumulative incidence estimates using Cox proportional hazards regression models and based on the presence of gout and the dual presence of gout and/or diabetes. The overall Cox proportional hazards regression model was adjusted for age, sex, race and ethnicity, body mass index, smoking history, and comorbidities (hypertension, cardiovascular disease, peripheral artery disease, cancer, cerebrovascular disease, chronic lung disease, dementia, diabetes, and kidney disease). The model of dual associations of gout and/or diabetes was adjusted for the same covariates with exception of diabetes. Shaded areas indicate 95% CIs.

Results based on gout and diabetes status are shown in Figure 2. In the absence of diabetes, patients with gout demonstrated a 1.56-fold increased rate (95% CI, 1.52-1.66) of LEA (eTable 4 in the Supplement). The highest rate was observed in patients with gout and diabetes (aHR, 3.36; 95% CI, 3.02-3.75), similar to the rate observed in those with diabetes alone (aHR, 3.21; 95% CI, 3.00-3.43).

In subsequent analyses limited to patients with gout, we examined associations of predefined measures of suboptimal ULT administration and suboptimal serum urate control with LEA. Groups characterized by suboptimal serum urate control, compared with those with adequate or indeterminate values for serum urate control and ULT administration, had universally higher LEA rates during the following year of observation (aHRs, 1.17-1.52) (Table 3). In contrast, suboptimal ULT did not appear to be associated with LEA occurrence. Frequencies in different groups at first observation were as follows: adequate or indeterminate serum urate and adequate or indeterminate ULT, 199 125 (40.2%); suboptimal serum urate and adequate or indeterminate ULT, 86 766 (17.5%); adequate or indeterminate serum urate and suboptimal ULT, 149 650 (30.2%); and suboptimal serum urate and suboptimal ULT, 59 591 (12.0%). Frequencies in different groups at last observation were as follows: adequate or indeterminate serum urate and adequate or indeterminate ULT, 194 439 (39.3%); suboptimal serum urate and adequate or indeterminate ULT, 53 917 (10.9%); adequate or indeterminate serum urate and suboptimal ULT, 211 519 (42.7%); and suboptimal serum urate and suboptimal ULT, 35 257 (7.1%).

**Table 3.** Associations of Serum Urate Control and Urate-Lowering Therapy Administration With LEA in Patients With Gout^a^

Variable	Any LEA	Toe	Transmetatarsal	Below knee	Above knee
Adequate or indeterminate serum urate and adequate or indeterminate ULT	1 [Reference]	1 [Reference]	1 [Reference]	1 [Reference]	1 [Reference]
Adequate or indeterminate serum urate and suboptimal ULT	0.95 (0.88-1.03)	0.94 (0.80-1.12)	0.96 (0.86-1.07)	0.95 (0.68-1.32)	0.94 (0.81-1.09)
Suboptimal serum urate and adequate or indeterminate ULT	1.37 (1.25-1.50)	1.39 (1.14-1.70)	1.31 (1.14-1.49)	1.45 (1.00-2.09)	1.44 (1.22-1.72)
Suboptimal serum urate and suboptimal ULT	1.26 (1.12-1.41)	1.52 (1.20-1.92)	1.20 (1.02-1.42)	1.20 (0.75-1.92)	1.17 (0.94-1.46)

Abbreviations: LEA, lower extremity amputation; ULT, urate-lowering therapy.

^a^ Adequate, suboptimal, and indeterminate serum urate levels and ULT are defined in the Methods section. For both ULT and serum urate control, adequate and indeterminate categories were combined into a single group for analyses. Patients categorized during each year of follow-up with associations with LEA in the following year were examined (all other variables were fixed at index value). All models were adjusted for age, sex, index calendar year, race and ethnicity, body mass index, smoking, hypertension, cardiovascular disease, peripheral vascular disease, cancer, cerebrovascular disease, lung disease, diabetes, and kidney disease.

## Discussion

In this cohort study of US veterans, we found that gout was associated with a 20% increase in the rate of LEA. This increase was independent of comorbidities, such as diabetes, CKD, peripheral vascular disease, and others that serve as established risk factors for LEA and are more common in gout. This finding has prognostic implications for patients with gout, with 5-year mortality approaching 70% in patients with diabetes after LEA and only slightly lower rates among those without diabetes.^26^ In addition to portending reduced survival, LEA is associated with worse physical functioning, lower health-related quality of life, and depression, with up to half of patients institutionalized in long-term care facilities postoperatively.^27,28^ Extrapolating to a general population that includes 9.2 million adults in the US with gout,^1^ our results suggest an approximately 2% cumulative incidence during a period of 15 years (Figure 2) and suggest that as many as 184 000 patients with gout are at risk of undergoing LEA during the coming years in the US alone. With gout and LEA frequency increasing in the VHA,^10,25^ an implication of these observations is that the burden posed to the health care system is likely to increase in this population over time.

In addition to being independent of comorbid conditions, our results found that the association of gout with LEA was higher in those without diabetes compared with the overall VA population. Although this may reflect the significantly lower risk of LEA among a comparator population without diabetes, it is also possible that recommended surveillance and standardized foot care applied in the setting of diabetes (but not routinely implemented in gout alone)^29^ might lead to reductions in LEA attributable to gout among those with both conditions. In patients with diabetes, structured foot care services have been shown to reduce the odds of LEA by more than 50%.^30^

Whether a proportion of LEA procedures in gout are preventable, similar to what has been shown in diabetes,^30^ is unknown. The association of LEA with suboptimal urate control should not be interpreted as causal because serum urate control could serve as a surrogate for gout severity or of other unmeasured or inadequately measured cofactors, such as severity of diabetes or CKD. The association of serum urate control is juxtaposed to results showing no association between attributes of ULT administration and LEA. These null findings may reflect real-world health care practices, which rarely involve ULT dose escalation and infrequent achievement of serum urate goals^31^ as recommended in management guidelines.^5,6^ A prior histopathologic survey^19^ showing gout as an unrecognized culprit for LEA, masquerading in preoperative assessments as nonhealing wounds or deep tissue infections, suggest that at least some amputations could be avoided. For instance, systematic screening for gout and/or hyperuricemia in patients being considered for LEA could identify patients who might benefit from noninvasive imaging studies, such as musculoskeletal ultrasonography or dual energy computed tomography, which can help to discriminate gout from its mimics.^32^ Our results, however, do not support tophaceous deposition as the sole explanation for excess amputations that occur in gout. The risk of LEA attributable to gout in this study was higher for below-the-knee than for toe or transmetatarsal procedures, with the toe or transmetatarsal being more common anatomical locations affected by tophus formation.^33^ Likewise, poor serum urate control, which could serve as a marker of tophaceous burden, rendered similar associations with LEA across procedure types. Whether implementation of systematic screening for gout in patients being considered for amputation or patient and health care professional education would improve long-term outcomes is unknown and will require further study.

### Limitations

This study has limitations. Inherent in its observational design and reliance on administrative data, the possibility of bias from misclassification (of exposure, outcome, and covariates), missing data, and loss to follow-up exists. To reduce misclassification, we required 2 or more gout diagnoses separated in time. In addition, prior work^7,34,35^ from our group demonstrated rates of ULT dispensing, comorbidity, and mortality that were similar to values reported in separate non-VHA gout populations. Likewise, sensitivity analyses limited to individuals without missing data and censoring with a loss of follow-up that exceeded 1 year suggest that our results are robust and not meaningfully affected by these potential sources of bias. Although we adjusted for relevant comorbid conditions, we were unable to assess the severity of these comorbidities, which may have resulted in residual confounding. Furthermore, comorbidities were assessed at baseline, but their status and severity may change over time. It is also possible that findings in the VHA may not be generalizable to other populations. However, as the largest integrated health system in the US with a patient population enriched for gout and related cardiometabolic conditions, the VHA provides an ideal context for examining health outcomes in gout.

## Conclusions

This study found that US veterans with gout were 20% more likely to undergo LEA than those without gout. This increase was independent of comorbidities and other risk factors and was apparent across different LEA types. Among those with gout, suboptimal serum urate control was associated with a higher rate of LEA. Further investigation is needed to understand the indications for LEA procedures conducted in gout in addition to identifying potential means of prevention as a way of ultimately improving long-term outcomes in this population.

## Notes

Supplement. **eTable 1.** *CPT-4*, *ICD-9-PCS*, and *ICD-10-PCS* Codes Used to Define Lower Extremity Amputation (LEA) by Site **eTable 2.** Diagnostic Codes Used for Identification of Chronic Conditions **eTable 3.** Full Multivariable Cox Models Demonstrating Associations of Gout and Other Patient Characteristics With Lower Extremity Amputation (LEA), Overall and by LEA Type **eTable 4.** Associations of Gout and/or Diabetes With Lower Extremity Amputation **eFigure.** Study Design Schema
