# Supplementary material for: Comparison of Rates of Lower Extremity Amputation in Patients With and Without Gout in the US Department of Veterans Affairs Health System
Source: JAMA Netw Open. 2022 Jan 6;5(1):e2142347. doi: 10.1001/jamanetworkopen.2021.42347 (PMC8739736; doi:10.1001/jamanetworkopen.2021.42347)
Supplement: Supplement. — eTable 1. CPT-4, ICD-9-PCS, and ICD-10-PCS Codes Used to Define Lower Extremity Amputation (LEA) by Site eTable 2. Diagnostic Codes Used for Identification of Chronic Conditions eTable 3. Full Multivariable Cox Models Demonstrating Associations of Gout and Other Patient Characteristics With Lower Extremity Amputation (LEA), Overall and by LEA Type eTable 4. Associations of Gout and/or Diabetes With Lower Extremity Amputation eFigure. Study Design Schema [file jamanetwopen-e2142347-s001.pdf]

Supplementary Online Content

Mikuls TR, Soto Q, Petro A, et al. Comparison of rates of lower extremity amputation in patients with and without gout in the US Department of Veterans Affairs Health System. *JAMA Netw Open*. 2022;5(1):e2142347. doi:10.1001/jamanetworkopen.2021.42347

eTable 1. *CPT-4*, *ICD-9-PCS*, and *ICD-10-PCS* Codes Used to Define Lower Extremity Amputation (LEA) by Site

eTable 2. Diagnostic Codes Used for Identification of Chronic Conditions

eTable 3. Full Multivariable Cox Models Demonstrating Associations of Gout and Other Patient Characteristics With Lower Extremity Amputation (LEA), Overall and by LEA Type

eTable 4. Associations of Gout and/or Diabetes With Lower Extremity Amputation

eFigure. Study Design Schema

This supplementary material has been provided by the authors to give readers additional information about their work.

© 2022 Mikuls TR et al. *JAMA Network Open.*

eTable 1: CPT-4, ICD-9-PCS and ICD-10-PCS codes used to define lower extremity amputation (LEA) by site [a]

Amputation Type	CPT-4	ICD-9-PCS	ICD-10-PCS
Toe	28825	84.11	0Y6P0Z0, 0Y6P0Z1, 0Y6P0Z2, 0Y6P0Z3, 0Y6Q0Z0, 0Y6Q0Z1, 0Y6Q0Z2, 0Y6Q0Z3, 0Y6R0Z0, 0Y6R0Z1, 0Y6R0Z2, 0Y6R0Z3, 0Y6S0Z0, 0Y6S0Z1, 0Y6S0Z2, 0Y6S0Z3, 0Y6T0Z0, 0Y6T0Z1, 0Y6T0Z2, 0Y6T0Z3, 0Y6U0Z0, 0Y6U0Z1, 0Y6U0Z2, 0Y6U0Z3, 0Y6V0Z0, 0Y6V0Z1, 0Y6V0Z2, 0Y6V0Z3, 0Y6W0Z0, 0Y6W0Z1, 0Y6W0Z2, 0Y6W0Z3, 0Y6X0Z0, 0Y6X0Z1, 0Y6X0Z2, 0Y6X0Z3, 0Y6Y0Z0, 0Y6Y0Z1, 0Y6Y0Z2, 0Y6Y0Z3, 0Y6M0Z9, 0Y6M0ZB, 0Y6M0ZC, 0Y6M0ZD, 0Y6M0ZF, 0Y6N0Z9, 0Y6N0ZB, 0Y6N0ZC, 0Y6N0ZD, 0Y6N0ZF
Transmetatarsal	28800, 28805, 28810, 28820	84.12	0Y6M0Z4, 0Y6M0Z5, 0Y6M0Z6, 0Y6M0Z7, 0Y6M0Z8, 0Y6N0Z4, 0Y6N0Z5, 0Y6N0Z6, 0Y6N0Z7, 0Y6N0Z8
Below knee	27880-1, 27888, 27889	84.13-84.15	0Y6M0Z0, 0Y6N0Z0, 0Y6H0Z1, 0Y6H0Z2, 0Y6H0Z3, 0Y6J0Z1, 0Y6J0Z2, 0Y6J0Z3
Above knee	27882, 27884, 27886, 27295, 27590-2, 27596, 27598	84.16-84.19	0Y6F0ZZ, 0Y6G0ZZ, 0Y6C0Z1, 0Y6C0Z2, 0Y6C0Z3, 0Y6D0Z1, 0Y6D0Z2, 0Y6D0Z3, 0Y670ZZ, 0Y680ZZ, 0Y620ZZ, 0Y630ZZ, 0Y640ZZ

a – Adapted from Cai et al. JAMA Network Open 2021;4(1)e2033953 [10]

© 2022 Mikuls TR et al. *JAMA Network Open.*

**eTable 2:** Diagnostic codes used for identification of chronic conditions	
**Condition**	**ICD-9 Codes**
Cancer	140.x–172.x, 174.x–208.x, V10.60-10.63, V10.69, V10.71-V10.72, V10.79
Cerebrovascular disease	362.34, 430.x–438.x
Chronic lung disease (Asthma, COPD, or ILD)	493.x (excluding 493.81), 491.x-492.x, 496.x, 515.x, 516.3, 516.8-516.9, 714.8
Coronary artery disease	410.x-414.x, 429.7, V45.81, V45.82
Dementia	290.x, 294.1, 331.2
Diabetes	250.x
Hypertension	401.x, 402.x-405.x
Peripheral vascular disease / aneurysm	093.0, 437.3, 440.x, 441.x, 443.1-443.9, 447.1, 557.1, 557.9, V43.4
Renal disease	403.01, 403.11, 403.91, 404.02, 404.03, 404.12, 404.13, 404.92, 404.93, 582.x, 583.0–583.7, 585.x, 586.x, 588.0, V42.0, V45.1, V56.x

© 2022 Mikuls TR et al. *JAMA Network Open.*

eTable 3: Full multivariable Cox models demonstrating associations of gout and other patient characteristics with lower extremity amputation (LEA), overall and by LEA type [a]

	**Any LEA**	**Toe**	**Transmetatarsal**	**Below knee**	**Above knee**
**Gout**	1.20 (1.16-1.24)	1.27 (1.18-1.37)	1.11 (1.06-1.16)	1.59 (1.39-1.81)	1.22 (1.15-1.30)
Demographics					
Age, years	0.99 (0.98-0.99)	0.99 (0.99-0.99)	0.98 (0.98-0.99)	0.96 (0.96-0.97)	0.99 (0.99-0.99)
Male sex (vs. female)	1.28 (1.09-1.50)	0.78 (0.58-1.06)	1.95 (1.45-2.61)	1.55 (0.80-2.98)	1.05 (0.80-1.39)
Race/ethnicity, %					
Black Non-Hisp	Ref.	Ref.	Ref.	Ref.	Ref.
Hispanic/Latinx	0.77 (0.72-0.82)	0.61 (0.52-0.72)	1.05 (0.98-1.14)	0.42 (0.32-0.56)	0.44 (0.38-0.51)
Missing	0.42 (0.40-0.44)	0.38 (0.33-0.43)	0.45 (0.42-0.49)	0.29 (0.24-0.36)	0.43 (0.39-0.48)
Other	0.81 (0.76-0.86)	0.72 (0.61-0.85)	0.98 (0.90-1.07)	0.51 (0.39-0.67)	0.65 (0.57-0.75)
White Non-Hisp	0.80 (0.78-0.83)	0.90 (0.84-0.97)	0.81 (0.77-0.84)	0.54 (0.48-0.61)	0.81 (0.76-0.86)
Health factors & comorbidity					
Body mass index					
<20 kg/m^2	1.73 (1.58-1.89)	1.52 (1.21-1.91)	2.00 (1.75-2.27)	1.73 (1.24-2.43)	1.48 (1.25-1.74)
20 to <25 kg/m^2	Ref.	Ref.	Ref.	Ref.	Ref.
25 to <30 kg/m^2	0.85 (0.81-0.89)	0.85 (0.76-0.95)	0.86 (0.81-0.93)	1.01 (0.85-1.19)	0.80 (0.74-0.87)
≥30 kg/m^2	1.02 (0.98-1.07)	1.13 (1.02-1.26)	1.05 (0.98-1.12)	1.05 (0.89-1.24)	0.94 (0.87-1.01)
Smoking,					
Never	Ref.	Ref.	Ref.	Ref.	Ref.
Former	1.39 (1.33-1.45)	1.14 (1.04-1.25)	1.19 (1.12-1.26)	1.43 (1.17-1.75)	2.11 (1.94-2.30)
Current	1.91 (1.84-1.99)	1.57 (1.44-1.72)	1.70 (1.61-1.80)	3.19 (2.65-3.84)	2.50 (2.30-2.72)
Missing	0.85 (0.78-0.93)	0.92 (0.77-1.10)	0.88 (0.78-0.99)	2.69 (2.04-3.55)	0.45 (0.35-0.56)
Hypertension	1.17 (1.14-1.21)	1.17 (1.09-1.24)	1.03 (0.99-1.08)	1.12 (1.00-1.25)	1.40 (1.33-1.48)
Cardiovascular disease	1.36 (1.32-1.40)	1.33 (1.25-1.42)	1.16 (1.12-1.21)	1.60 (1.42-1.80)	1.74 (1.65-1.83)
Peripheral artery disease	2.31 (2.22-2.39)	2.21 (2.02-2.41)	2.98 (2.84-3.13)	1.85 (1.54-2.21)	1.44 (1.33-1.56)
Cancer	1.19 (1.15-1.24)	1.23 (1.12-1.34)	1.08 (1.03-1.14)	1.46 (1.25-1.70)	1.31 (1.20-1.37)

© 2022 Mikuls TR et al. *JAMA Network Open.*

Cerebrovascular disease	1.35 (1.30-1.41)	1.39 (1.26-1.53)	1.35 (1.27-1.43)	1.33 (1.10-1.61)	1.36 (1.23-1.41)
Chronic lung disease	1.15 (1.11-1.19)	1.20 (1.10-1.30)	0.94 (0.89-0.99)	1.30 (1.12-1.50)	1.50 (1.41-1.59)
Dementia	1.21 (1.06-1.38)	1.25 (0.91-1.72)	0.96 (0.77-1.18)	1.72 (1.01-2.92)	1.55 (1.24-1.94)
Diabetes	3.27 (3.18-3.35)	2.96 (2.78-3.14)	6.19 (5.95-6.44)	1.21 (1.08-1.36)	1.47 (1.40-1.55)
Renal disease	1.67 (1.61-1.75)	1.70 (1.54-1.87)	1.91 (1.81-2.02)	1.20 (0.97-1.50)	1.35 (1.24-1.48)

a – “Other” category for race/ethnicity comprised of American Indian or Alaska Native, Asian, multiple race, or Native Hawaiian or other Pacific Islander.

© 2022 Mikuls TR et al. *JAMA Network Open.*

eTable 4: Associations of Gout and/or Diabetes With Lower Extremity Amputation

	aHR (95% CI)	*P* value
No gout and no diabetes	1 (reference)	NA
Gout and no diabetes	1.56 (1.41-1.72)	<0.001
Diabetes and no gout	3.21 (3.00-3.43)	<0.001
Gout and diabetes	3.36 (3.02-3.75)	<0.001

Models adjusted for age, sex, race/ethnicity, body mass index (BMI, kg/m^2), smoking history, and comorbidities (hypertension, cardiovascular disease, peripheral artery disease, cancer, cerebrovascular disease, chronic lung disease, dementia, and renal disease).

Abbreviations: aHR, adjusted hazard ratio; CI, confidence interval

© 2022 Mikuls TR et al. *JAMA Network Open.*

eFigure 1.

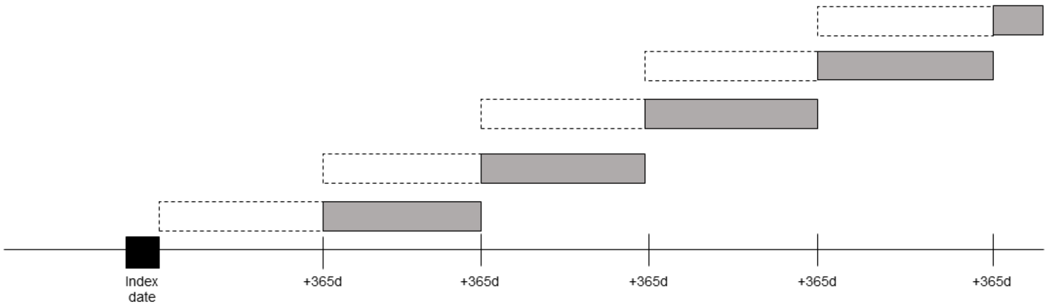

Gout management assessment period evaluating urate lowering therapy and laboratory-based serum urate concentrations. If serum urate was missing, values from the preceding year were included.

Gout management categorization based on prior assessment period. Patients categorized as adequate/indeterminate vs. suboptimal urate lowering therapy and serum urate control.

© 2022 Mikuls TR et al. *JAMA Network Open.*

© 2022 Mikuls TR et al. *JAMA Network Open.*
